# The Myth of The Annular Lipids

**DOI:** 10.3390/biomedicines10112672

**Published:** 2022-10-22

**Authors:** Juan C. Gómez-Fernández, Félix M. Goñi

**Affiliations:** 1 Department of Biochemistry and Molecular Biology (A), Faculty of Veterinary Science, Universidad de Murcia, 30100 Murcia, Spain; jcgomez@um.es; 2 Instituto Biofisika (CSIC, UPV/EHU), Department of Biochemistry, University of the Basque Country, 48940 Leioa, Spain

**Keywords:** lipid annulus, annular lipid, boundary lipid, intrinsic membrane proteins, lipid-protein interactions, resonance spectroscopies

## Abstract

In the early 1970s, the existence of a “lipid annulus” stably surrounding the individual intrinsic protein molecules was proposed by several authors. They referred to a number of lipid molecules in slow exchange with the bulk lipid in the bilayer, i.e., more or less protein-bound, and more ordered than the bulk lipid. The annular lipids would control enzyme activity. This idea was uncritically accepted by most scientists working with intrinsic membrane proteins at the time, so that the idea operated like a myth in the field. However, in the following decade, hard spectroscopic and biochemical evidence showed that the proposed annular lipids were not immobilized for a sufficiently long time to influence enzyme or transporter activity, nor were they ordered by the protein. Surprisingly, forty years later, the myth survives, and the term ‘annular lipid’ is still in use, in a different, but even more illogical sense.

## 1. Introduction and Disambiguation

The early 1970s saw the advent of the concept of intrinsic, or integral, membrane proteins, and the possibility of purifying them in active form, usually in the presence of detergents. Integral protein reconstitution in bilayers of defined lipid composition was also made possible in those years, leading to studies on the role of lipids in protein structure and function, what was called the problem of lipid-protein interaction. All these truly seminal discoveries were taking place in the frame of the famous Singer and Nicolson paper [1], whose half-century this review intends to commemorate.

The existence of a “lipid annulus” stably surrounding the individual intrinsic protein molecules was proposed by several authors in 1973–1976 [2,3,4,5,6,7]. Annular lipid was often called “boundary lipid”, and, more rarely, “halo lipid”. These three denominations should be considered as synonym. They referred to a number of lipid molecules in slow exchange with the bulk lipid in the bilayer, i.e., more or less protein-bound, and more ordered than the bulk lipid, as detailed below. A word of disambiguation will be useful when conducting literature searches on this topic. *Annulus* is the Latin name for ‘ring’; thus, it is not surprising that it is very commonly found referring to ring-like structures in all kingdoms of life, most often without reference to membrane lipids. Boundary is a common word used as a synonym of border, frontier, or limit; thus, it is also often found in biology without specific reference to membranes. It may be confusing, however, that, when lateral phase separation occurs in a bilayer or monolayer, the dividing line between phases is usually designated as the ‘phase boundary’, totally unrelated to the presence or absence of proteins in the system.

In the present contribution, the term ‘myth’ is used according to the second meaning in Merriam-Webster’s:

*2, a popular belief or tradition that has grown up around something or someone.*

It is relevant, in this respect, the view of the great Danish scholar Thorkild Jacobsen, that “a myth never has one meaning only”, and also that “a myth is a polyphonic fugue of many voices”. This paper summarizes the process of conversion of a scientific proposal into a myth that survives to our day, together with the development of new fugal voices, sometimes discordant.

## 2. 1973: The Annulus Is Born

Perhaps the first paper in which the existence of boundary, or annular, lipids was proposed was published by Jost et al. in February 1973 [2]. This article also inaugurated what would become a common framework for annulus studies in the years to follow. In this sort of studies an integral protein would be purified (cytochrome c oxidase from beef heart mitochondria in [2]) and reconstituted at different lipid/protein ratios. Then a fatty acid (or a phospholipid) containing a spin label would be added, and the behavior of this probe examined by electron spin resonance (ESR), also called electron paramagnetic resonance (EPR), spectroscopy. Two spectral components were typically observed, corresponding to two probe populations, respectively, more and less mobile. As it happened, a high degree of immobilization was often found at a lipid/protein ratio corresponding to a single layer of phospholipid surrounding the protein. The data were interpreted as evidence for a boundary of immobilized lipid between the hydrophobic protein and adjacent fluid lipids in the bilayer [2].

It should be noted that the boundary as such was never observed, and that the existence of the annular lipids was inferred from the coincidence between the immobilized lipid fraction and the presumed transmembrane perimeter of the enzyme. This did not deter a high number of scientists from uncritically accepting the annulus and embarking on the discovery of immobilized boundary lipids in their study systems, as, in fact, they did. To cite but a few examples, a “quasi-crystalline” phospholipid halo would enclose the liver microsome cytochrome P450 reductase, while the bulk lipid of the membrane was in a “rather fluid state” [3]; an annulus of at least 30 lipid molecules would surround sarcoplasmic reticulum Ca^2+^-ATPase reconstituted in dipalmitoyl phosphatidylcholine bilayers [4], and lipophilin, a hydrophobic protein from myelin, would also give rise to an immobilized lipid fraction, “presumably due to the presence of boundary lipid around the protein” [5]. Put in historical perspective, it is remarkable the general and undisputed acceptance of the existence of a lipid annulus as a genuine principle of membrane organization, when only indirect evidence had been produced for it.

Once the existence of such an annulus was generally accepted, further properties were assigned to it, again in the absence of sound experimental evidence. It was ‘demonstrated’ that cholesterol was excluded from the phospholipid annulus surrounding an active Ca^2+^-ATPase [6], in turn the ‘fact’ that cholesterol was excluded was often invoked as a proof of the existence of the annulus. Annular lipids would exist in the gel phase [7] and local anesthetics would trigger a change of the annular lipid from the gel to the liquid-crystalline state, with a consequent relaxation of the sodium channel to an inactive configuration [7]. Fatty acid chain length between C14 and C18 would not greatly influence boundary lipid formation [8], a somewhat unexpected observation of a putative lipid annulus covering the hydrophobic, transmembrane part of an intrinsic protein.

## 3. The Critical Voices

Within that flood of annuli, a few voices were heard of scientists swimming against the tide. In 1978, Marsh and Barrantes [9] used the ESR technique to study the immobilized lipid in acetylcholine receptor-rich membranes from *Torpedo marmorata*. They found that the proportion of lipid in the immobilized component was greater than calculated for a single boundary layer around the protein, and that it would rather correspond to the total interstitial lipid occupying the area between densely packed protein units in acetylcholine receptor-rich membranes. Shortly afterwards, Beauregard and Roufogalis [10] studied the cardiolipin that was found associated with acetylcholine esterase when this enzyme was purified from bovine erythrocytes and concluded that cardiolipin was bound to the core of the dimeric protein structure, rather than forming a boundary layer. In fact, this was one of the early demonstrations of a specific lipid playing a structural role in a protein [11]. Litman et al. [12], studying the meta I to meta II transition in rhodopsin, found no evidence for the formation of a phospholipid boundary layer around rhodopsin.

Nuclear magnetic resonance (NMR) spectroscopy happened to be an enormously powerful technique when applied to lipid-protein interactions. Brown et al. [13] used ^1^H-NMR to study retinal rod outer membranes, with the result that a single component associated with lipid signals could be detected. Two other important papers containing NMR data were published towards the end of 1978 [14,15]. Oldfield et al. [14] applied deuterium NMR to the study of mixtures of integral proteins with selectively deuterated dimyristoyl phosphatidylcholine, at temperatures above and below the T_c_ gel-to-fluid phase transition temperature of the lipid. In contrast with the previous ESR studies, with lipids in the fluid phase, NMR did not reveal any ordered boundary lipid. Below the T_c_, the lipid hydrocarbon chains were prevented from crystallizing by the protein. Similar disordering effects above T_c_ were seen with an unsaturated, deuterated phosphatidylcholine and cytochrome c oxidase. Seelig and Seelig [15] applied deuterium and 31-phosphorus NMR to study systems containing selectively deuterated POPC and cytochrome c oxidase. They found that incorporation of the enzyme into PC bilayers led to a more disordered conformational state of the lipids, due to the irregular protein surface that was sensed by all lipid molecules. These measurements did not suggest any special type of boundary lipid.

Soon afterwards, further contributions by Oldfield and co-workers [16,17] confirmed and extended the previous NMR investigations to systems containing sarcoplasmic reticulum Ca^2+^-ATPase, human brain lipophilin, beef brain myelin proteolipid apoprotein, or cytochrome c oxidase, reconstituted in selectively deuterated DMPC or DPPC. They found that proteins either disordered or had little effect on hydrocarbon chain order in membranes in the fluid state. No evidence for any ordered boundary lipid in association with the protein was found above T_c_, which the authors attributed to the rough nature of protein surfaces. In the fluid bilayers, exchange between free bilayer and protein-associated lipid was fast on the time scale of the deuterium NMR experiment (≥10^3^ s^−1^).

A different line of experimentation, based on fluorescence quenching, was used by Feigenson and co-workers to examine critically the annulus proposal, in reconstituted Ca^2+^-ATPase bilayers. The method was developed by London and Feigenson [18]. They utilized a nitroxide spin-labeled phosphatidylcholine to quench the fluorescence from a variety of membrane-bound molecules by a static process. The distance dependence of the fluorescence quenching of diphenylhexatriene, p-terphenyl, and molecules containing tryptophan arose only from spin-labeled phospholipids in contact with the fluorophore [18]. Using this method, London and Feigenson were able to obtain phospholipid binding constants to the Ca^2+^ATPase from an analysis of fluorescence quenching data [19]. The binding constants for a number of phospholipid species were nearly identical when the phospholipids were in the liquid-crystalline state. However, temperature or Ca^2+^-induced phase separation of phospholipid led to striking changes in the composition of the phospholipids in contact with the Ca^2+^ATPase, relative to the overall composition of the membranes. In the context of the boundary lipid proposal, the technique was applied to discern whether those lipids in contact with protein differed from free lipids with respect to either lipid composition or local motion and order. Fluorescence quenching indicated that, at neutral pH, for the bulk of the binding sites, the Ca^2+^ATPase did not selectively bind phospholipids [19]. Furthermore, Caffrey and Feigenson found, using again the above method, that the Ca^2+^ATPase bound with equal affinity phosphatidylcholines regardless of fatty acyl chain length or details of unsaturation, from dodecenoyl (C12:1) to nervonoyl (C24:1) [20]. This was against the expectations of the annulus proposal, according to which the protein would preferentially bind those phospholipids whose length adjusted to the hydrophobic surface of the protein in contact with the membrane matrix.

## 4. A Unified Explanation

The apparent contradiction between ESR and NMR spectroscopic data, plus a number of other observations that had been interpreted for and against the existence of a lipid annulus, were critically examined by Dennis Chapman and co-workers. In 1977–1982, they produced a unified explanation, based on their own and many other experimental data, which did not require the existence of a ‘boundary lipid’, at least in the form that had been accepted in the previous years. Their 1979 review [21] contains most of these ideas.

**Figure 1:**
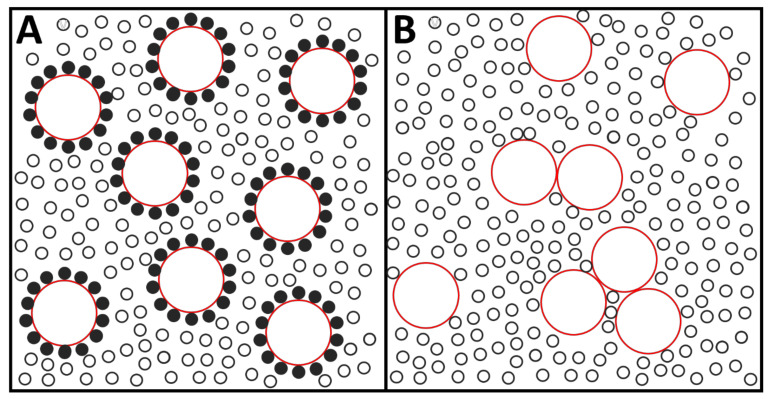
An outline of two views of protein-lipid interactions. (**A**), the annulus view. (**B**), the random distribution view. Circles in red: proteins. Black circles: lipids. Filled black circles: “annular lipids”. Entropic factors clearly favor the disposition in (**B**). The figure is extremely simplified. Note, in particular, the unnatural smooth perimeter of the proteins. Total lipid:protein ratio, 32:1. (Original from Dr. J. Sot).

(a) The apparent contradiction between ESR and NMR could be due to the different motional rates necessary to produce averaging of the spectra in the two cases. Since the NMR frequencies are less than those in ESR spectroscopy, the distinct components seen with spin-label ESR could be averaged over the much longer NMR time scale and only a single component observed. The corresponding time scales would typically be of 10^−8^ s for ESR and of 10^−3^–10^−5^ s for NMR. Thus, a probe could be immobile for 10^−8^ s, and detected as such by ESR, but exchange freely with the neighboring molecules at the 10^−3^–10^−5^ s time scale, so that NMR would detect a single population of probes. This point was stressed, some years later, by Brown and co-workers [22], on the basis of ^31^P-NMR studies.
(b) The above explanation, however, left unanswered the functional implications of the resonance spectroscopy data. One of the tenets of the boundary lipid proposal was that these lipids determined the enzyme activity of the encircled protein [4,7]. In principle, it could be assumed that an immobile annulus with an average life of 10^−8^ s could somehow influence the enzyme activity. But, in fact, the turnover time, i.e., the time required for a single catalytic cycle, of most intrinsic membrane enzymes, including the transport ATPases and the respiratory chain H^+^-bombs, such as cytochrome c oxidase, is typically in the 10^−2^–10^−3^ s range [23,24]. In consequence, enzyme activity cannot be expected to be modified by lipid events occurring with time lengths shorter by 5 or 6 orders of magnitude.
(c) Various intended demonstrations of the lipid annulus were based on the observation that, when a lipid-protein system was gradually delipidated, the enzyme activity fell precipitously below a given lipid/protein ratio [4,6,25], and/or a spectral signal of immobilized lipid became apparent [2,4,26,27]. This critical lipid/protein ratio would be an indication of the boundary lipid existing in that particular system. The idea behind this rationale appeared to be that protein-protein contacts could not occur without a high probability of irreversible protein denaturation, which would be prevented by the protecting annulus. Preventing contacts would compensate the entropic effort of organizing the boundary lipids versus allowing random mixing of the membrane components. These two views are outlined in Figure 1. Lipids and proteins are represented there as small and large circles, respectively, at a 32:1 mol ratio. In A, each protein is surrounded by 14 lipids, representing the ‘annulus view’. B corresponds to the ‘random mixing view’. A clear entropy difference, favoring B, is apparent. The authoritative 2005 review by Engelman [28] offers a model that, in agreement with Singer and Nicolson [1], corresponds to a random mixing of components, and, on the basis of evidence accumulated in the two decades elapsed between both papers, includes extensive protein-protein contacts. Such contacts had been proposed by Chapman and co-workers [21]. Furthermore, the latter authors wrote: “Some authors have commented upon the fact that a minimum amount of lipid is required for optimum enzymatic activity and attempted to relate this to the presence of a special lipid annulus. However, a minimum amount of lipid may be required merely to retain the appropriate fluidity for the protein to operate…”. A related study with Ca^2+^-ATPase reconstituted in DPPC [25] described how, for a lipid:protein mol ratio at or above 50:1, freeze-fracture electron microscopy of samples below 25 °C revealed that the proteins aggregated into patches, leaving aside areas of pure lipid. The latter, which was in the gel phase, started to melt at 28–30 °C and, at the same time, protein rotational motion begun to occur, together with a marked increase in enzyme activity. Thus, in this system, protein aggregation did not lead to irreversible denaturation.
(d) An additional important aspect of the proposed annular lipids was that they would be more ordered than the surrounding fluid lipids, the increased order arising from the long contact with the protein. In fact, proteins are not the smooth cylinders depicted in many cartoons, but rather present highly irregular surfaces, covered by a large variety of amino acid side chains. Using the then novel application of IR spectroscopy to aqueous systems, Chapman and co-workers [29] showed that, except at very high (unphysiological) lipid:protein ratios, the presence of intrinsic proteins would not change, or would even increase, the proportion of *gauche* conformers in the fatty acyl chains, i.e., lipid order would remain unchanged, or decrease as a result of protein insertion.

In summary, the available experimental evidence argued against the existence of long-lived, ordered annuli surrounding the transmembrane domain of intrinsic proteins and controlling their activities. Instead, proteins and lipids would diffuse at random in the plane of the membrane, direct lipid-protein contacts being too brief to influence protein function beyond the overall effects of the lipid environment and membrane fluidity. This conclusion would not deny the existence of specific lipid binding to certain proteins [11], nor would it contradict the limitations on protein lateral motion imposed by ‘membrane domains’ [28,30], whose existence was hardly suspected when the above studies were performed.

## 5. Birth and Perpetuation of a Myth

As discussed in the preceding sections, the decade between 1973 and 1983 saw the proposal of the existence of a lipid annulus, its rapid propagation, and its experimental refutation. It must be stressed that the seminal experiments giving rise to the idea of a boundary lipid were technically correct (albeit the interpretations were far-fetched or even flawed), and that no hint of a suspicion of improper behavior of the authors involved can be contemplated. Moreover, it was totally out of the reach of those authors the fact that the lipid annulus would become a myth, in the sense discussed in the Introduction. Why this happened, its reasons and mechanisms, are beyond the scope of this review, and certainly beyond the intellectual capacity of its authors. Suffice it to say at this point that similar myths have occurred in the not-so-distant past in the field of membrane biophysics, e.g., the ‘phosphorylated intermediate’ of mitochondrial oxidative phosphorylation, the protein ‘molten globule’, or the ‘lipid rafts’.

An anonymous referee of this paper, to whom the authors are sincerely grateful, suggests that the origin and permanence of scientific myths may have a linguistic basis, an easily remembered term like “annulus” (or “lipid raft”) being quickly picked up and repeated, often by people somewhat peripheral to the field itself. Dawkins’ concept of “meme” may be relevant in this context. *Britannica* defines meme as a “unit of cultural information spread by imitation”. The term was introduced in 1976 by British evolutionary biologist Richard Dawkins in his work, *The Selfish Gene* [31]. Dawkins conceived of memes as the cultural parallel to biological genes and considered them, in a manner similar to “selfish” genes, as being in control of their own reproduction and thus serving their own ends.

A more contemporary term, applicable to many areas in science, would be “clickbait”, defined by Wikipedia as a text or a thumbnail link that is designed to attract attention and to entice users to follow that link and read, view, or listen to the linked piece of online content. We can now understand how “annulus” worked, in the 70′s and 80′s of the past century, as an “avant la lettre clickbait”. For a formal philosophical treatment of memes and related language elements, see the Schaden and Patin’s 2017 discussion [32].

If the birth and propagation of a scientific myth are difficult to explain, its perpetuation against the experimental evidence is even more mysterious. The studies by Chapman, Oldfield and others, summarized above, were certainly effective in quenching the epidemic of lipid annuli, this sort of publication becoming rare after 1983. Studies accepting, either explicitly or implicitly, the ‘annulus-skeptical’ views began to appear [33,34,35,36]. It is, then, interesting to note (perhaps for the historian or sociologist of science, rather than for the scientist in the strict sense), that the annulus myth retains some signs of vitality forty years later. A literature survey, which may not be comprehensive, in August 2022 has delivered 26 papers in which annular lipids are mentioned, only in the decade 2013–2022. Virtually all of them have in common the loose, when not incorrect, use of the terms ‘boundary’ or ‘annular’ lipids. In many cases, e.g., [37,38], non-specific annular interactions are opposed to specifically bound lipids, in terms suggesting that only those two kinds of membrane lipids exist, i.e., it appears that annular lipids would correspond to the vast majority of lipids (the ‘bulk lipid’), in flagrant contradiction with the historical use of the term. Medina-Carmona et al. [39] are more specific, they distinguish between “non-annular lipids […] tightly bound to cavities in the hydrophobic regions of the protein [and] non-exchangeable”, and “Annular lipids [that] constitute the first layer of lipids surrounding the membrane-bound system and have restricted mobility compared to bulk lipids, but may exchange with the bulk lipids”, thus these authors go back to the 1970s [2,4] and ignore the refutation of the annulus achieved by NMR studies at the end of that decade [14,15]. In all those 26 papers, the 20th-century literature is ignored or misinterpreted, with the result of confusing or meaningless interpretations of the experimental results.

It is noteworthy that, in almost all of the 26 papers mentioned above, techniques that were not generally available in the past century were applied. Unfortunately, the novel techniques have not been accompanied by improved analyses of the results. Molecular dynamics (MD) is often used, e.g., [40,41]. However, the inherent limitations of the technique prevent running the experiments for longer than a few microseconds. Thus, MD leads to the same misinterpretations described above for ESR spectroscopy, namely that it describes the dynamics of lipid molecules in a time scale that is orders of magnitude below the time required for the average intrinsic protein to complete a single catalytic cycle (10^−2^–10^−3^ s). Other studies [37,42] have combined the use of mass spectrometry and detergent solubilization. In some cases, the membrane protein was gradually delipidated by timed exposure to a detergent [42], and the authors assumed that after prolonged delipidation, the remaining lipids would correspond to those in direct contact with the protein in the native conditions. However, even sub-solubilizing concentrations of detergents are known to perturb strongly the lipid bilayer (see, e.g., [43,44]), and there is no proof whatsoever that the most detergent-resistant lipid would be the one originally interacting with the protein.

Several recent papers in which the term ‘annular lipids’ is used include the use of styrene-maleic acid (SMA) to extract membrane proteins in the form of nanodiscs [45,46]. This methodology appears to respect the intrinsic protein conformation far better than the detergent solubilization-reconstitution method; thus, it is being used with success in the 3D-resolution of intrinsic proteins by NMR, or by cryo-electron microscopy. However, the situation is different when SMA nanodiscs are used, often in combination with mass spectrometry, in the study of lipid-protein interactions. The standard protocol for SMA nanodisc formation involves the joint incubation of membrane and polymer for several hours [47,48]. This time is many orders of magnitude longer than that of the phospholipid lateral diffusion in the plane of the membrane (diffusion coefficient of lipids is of the order of 10^−7^ cm^2^ s^−1^) [49,50]. Thus, the lipid composition of the SMA nanodiscs can in no way reflect the native membrane organization.

Atomic force microscopy (AFM) has also been used in the study of lipid-protein interactions [51,52], as well as electron crystallography of 2D-crystals [53]. In all cases, the misnomer ‘annular lipids’ is given to all the lipids that are not specifically bound to the protein.

Very recently, two interesting reviews have been published, dealing with lipid-protein interactions. Sych et al. [54] is focused on methodological aspects, and on specific (stoichiometric) lipid binding by membrane proteins. Levental and Lyman [55] offer a balanced description of the lipids in contact with intrinsic membrane proteins, concluding that “few of these lipids are ‘bound’ in any thermodynamically meaningful sense, in that they are rapidly replaced by other, often different, lipids from the bulk membrane”. This can be correlated with our 1982 review in [56].

## 6. Conclusions

Studies four-decades old demonstrated that the so-called ‘annular lipids’ or ‘boundary lipids’, surrounding an integral protein, were irrelevant for membrane function. This was so because, even if they were immobilized in contact with the protein, this occurred for a time length orders of magnitude shorter than the functionally relevant turnover time of the enzyme catalytic cycle. In other words, annular and bulk lipids would exchange thousands of times while the enzyme/transporter processed a single substrate molecule. In our days, the term ‘annular lipids’ has survived, but with a different meaning, now referring to the lipids that are not specifically bound to the protein, even if they have no interaction with the protein whatsoever. Examining the ‘annular lipid’ literature in the past decade, the quote from T. Jacobsen comes irresistible to the mind: “a myth never has one meaning only” [57]. Or, even better, as put by his fellow-Sumerologist S.N. Kramer, cited in [57], “the story seemed to make no connected sense; and what could be made out, seemed to lack intelligent motivation”.
